# Impact of Medicaid Expansion Under the Affordable Care Act on Receipt of Surgery for Breast Cancer

**DOI:** 10.1097/AS9.0000000000000194

**Published:** 2022-08-24

**Authors:** Leisha C. Elmore, Meng Li, Heather Lin, Yu Shen, Simona F. Shaitelman, Gildy Babiera, Nina Tamirisa, Isabelle Bedrosian

**Affiliations:** From the * Department of Breast Surgical Oncology, The University of Texas MD Anderson Cancer Center, Houston, TX; † Department of Health Services Research, The University of Texas MD Anderson Cancer Center, Houston, TX; ‡ Department of Biostatistics, The University of Texas MD Anderson Cancer Center, Houston, TX; § Department of Radiation Oncology, The University of Texas MD Anderson Cancer Center, Houston, TX; ∥ MD Anderson Physician Network, The University of Texas MD Anderson Cancer Center, Houston, TX.

**Keywords:** ACA, breast cancer, surgery

## Abstract

**Objective:**

To determine whether Medicaid expansion under the 2010 Affordable Care Act affected rates of breast cancer surgery.

**Background:**

Data regarding the impact of Medicaid expansion on access to surgical treatment of breast cancer are limited.

**Methods:**

Patients in the National Cancer Database diagnosed with non-metastatic breast cancer between January 1, 2010 and December 31, 2017 and residing in a state that expanded Medicaid in January 2014 or in a state that opted out of expansion were included. A quasi-experimental, difference-in-differences (DID) approach was used to assess rate of omission of surgical treatment.

**Results:**

Of 624,237 patients diagnosed with invasive breast cancer, 24,728 (4%) patients did not undergo surgical treatment. Overall, no significant differences in rates of omission of surgery over time were seen based on Medicaid expansion status. Significant findings were noted based on patient residential location. In rural areas, Medicaid expansion was associated with lower rates of omission of surgery (adjusted DID −2.47%, 95% confidence interval [CI] −4.01% to −0.94%; *P* = 0.002). In urban area, rates of omission of surgery increased over time for both groups, but the relative increase was lower in expansion states (adjusted DID −0.72%, 95% CI −1.25% to −0.20%; *P* = 0.007). In metro areas, changes in rates of surgery over time were comparable across expansion and non-expansion states (adjusted DID −0.08%, 95% CI −0.32% to 0.16%; *P* = 0.512).

**Conclusions:**

Medicaid expansion had no measurable effect on the receipt of surgery for breast cancer in the overall cohort. Medicaid expansion was associated with higher rates of surgery in rural areas, representing the minority of the population.

## INTRODUCTION

The 2010 Affordable Care Act (ACA) was a landmark policy that included expansion of Medicaid coverage, which resulted in a significant increase in eligibility for health insurance coverage for individuals in the United States. In 2012, a decision by the US Supreme Court allowed states to opt out of Medicaid expansion.^1^ This resulted in variable uptake across the United States, with some states choosing not to expand Medicaid coverage. Studies have demonstrated that the ACA has resulted in an increase in Medicaid coverage and a reduction in the rates of individuals without insurance in states that elected to adopt Medicaid expansion compared to non-expansion states.^2–4^

Since the implementation of the ACA, several studies have analyzed the impact of expanded Medicaid coverage on access to care and health outcomes. In patients with cancer, Medicaid expansion has been associated with an earlier stage at diagnosis.^5–7^ In a National Cancer Database (NCDB) analysis of patients with breast cancer, a lower incidence of advanced stage disease was seen in states adopting Medicaid expansion compared to those that opted out of expansion.^8^ While moving the needle on stage at diagnosis is a critical element for patients carrying a cancer diagnosis, this provides an incomplete window into the impact of the ACA on oncologic care. To date, there are limited data on the impact of Medicaid expansion on surgical management, particularly for patients with newly diagnosed, non-metastatic breast cancer, for whom surgery remains a critical component of treatment.

In a previous NCDB analysis evaluating barriers to surgical care in patients with breast cancer, our group noted that lack of insurance was independently associated with omission of surgery.^9^ In a separate population-based study of treatment variation by insurance status for breast cancer patients, uninsured women were less likely to receive surgical treatment for breast cancer.^10^ Nearly 13 million adults gained insurance with expansion of Medicaid and thereby gained enhanced access to and affordability of both preventative and curative care, including surgical care. This expanded access to care has resulted in more timely access to surgical care, improved quality of care as well as improved access to post-discharge care.^7^ Medicaid expansion has been associated with an increase in the use of outpatient surgical procedures^11^ and in breast cancer patients, a reduced incidence of advanced stage breast cancer.^8^ However, whether the increased access to care provided by Medicaid expansion mitigates the association seen between lack of insurance and omission of surgical therapy remains unknown. While in general the proportion of women who omit surgical care remains low, fully understanding the impact of policy change on all elements of cancer care remains important. Therefore, we sought to evaluate whether the increased access to care provided through Medicaid expansion impacted the receipt of surgery in patients with newly diagnosed invasive breast cancer. We hypothesized that Medicaid expansion would result in lower rates of omission of surgery in patients undergoing treatment for breast cancer.

## METHODS

### Data Source and Patient Population

**FIGURE 1.**
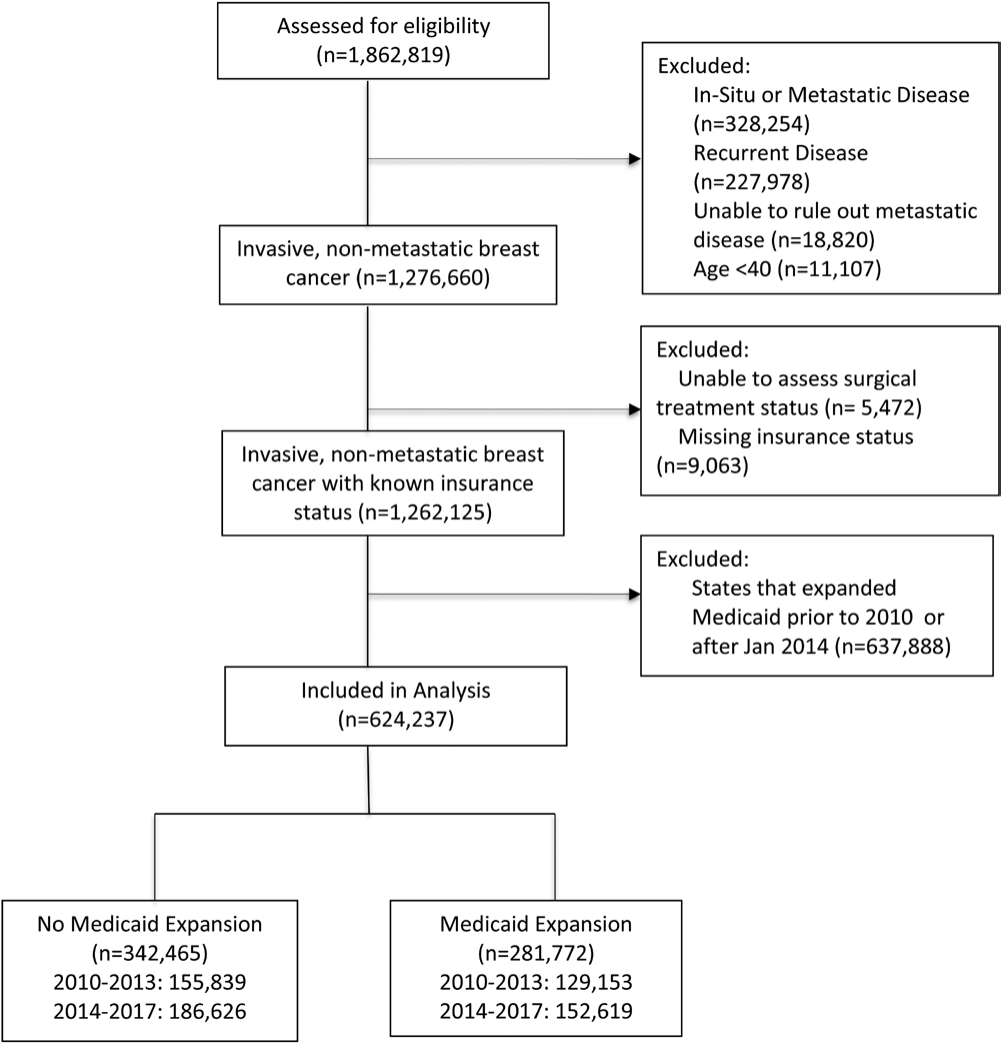
Consolidated standards of reporting trials flow diagram depicting selection process of patient cohort for analysis.

We queried the NCDB to extract a cohort of 624,237 patients with invasive breast cancer diagnosed between 2010 and 2017 from a population of 2,981,732 patients with breast cancer included in the database during the period of study. The NCDB is a national oncology outcomes database born from a quality initiative of the American College of Surgeons Commission on Cancer (CoC) and the American Cancer Society. More than 1500 CoC-accredited cancer programs in the United States and Puerto Rico report to the NCDB, and the database captures hospital-level data for approximately 70% of newly diagnosed cancer cases.^12^ Women with invasive breast cancer age 40 or older were included in the analysis. Patients were excluded if they had in situ or metastatic disease. Details regarding the stepwise exclusion of patients are shown in Figure 1. Given the relative infrequency of patients under age of 40, some sites may not have very many young breast cancer patients, therefore NCDB suppresses certain variables, including Medicaid expansion status, in order to avoid potential identification of such patients. Consequently, patients under the age of 40 were excluded from our analysis. Since breast cancer in women under 40 represents a small percentage of cases, this exclusion is unlikely to significantly bias our findings. In our study sample, only 1.7% of patients were under 40 (11,107 patients of a total of 624,237). We chose not to exclude older age women who would potentially be covered by Medicare because patients can be dual eligible for Medicare and Medicaid. In 2019, 19% of enrollees were dual enrolled. Thus, we included Medicare eligible patients in order to ensure we fully understood the impact of Medicaid expansion. Due to the absence of patient identifiers in the NCDB, our institutional review board deemed our analysis exempt from review and approval.

### Outcome Measures

The aim of the analysis was to evaluate the impact of Medicaid expansion on the primary outcome of rate of omission of surgery. Patient residence at time of diagnosis was used to stratify patients into non-expansion and Medicaid expansion states. Nineteen states were classified as non-expansion states (TN, NC, ID, GA, FL, MO, AL, MS, KS, TX, WI, UT, SC, SD, VA, OK, NE, WY, ME) and 19 states expanded Medicaid in January 2014 (KY, NV, CO, OR, NM, WV, AR, RI, AZ, MD, MA, ND, OH, IA, IL, VT, HI, NY, DE). Patients residing in states that expanded Medicaid prior to or after January 2014 were excluded from the analysis. In order to evaluate the impact of Medicaid expansion, patients were stratified into 2 time periods based on date of diagnosis. The pre-Medicaid expansion period included patients diagnosed from 2010 to 2013 and the post-Medicaid expansion period included patients diagnosed from 2014 to 2017.

### Statistical Analysis

Descriptive statistics were used to summarize sociodemographic, clinicopathologic, and treatment information. Chi-squared tests for categorical variables and *t* test\ANOVA or the counterparts of the non-parametric approaches (Wilcoxon rank-sum or Kruskal-Wallis) for continuous variables were used to compare patient and facility characteristics between patients residing in expansion and non-expansion states during the pre- and post-expansion time periods, respectively.^13^

#### Difference-in-Differences Analysis

The difference-in-differences (DID) approach was applied to evaluate the association between change in percentages of patients with omission of surgery over time and the ACA Medicaid expansion status.^14^ In this approach, a linear regression model was employed to the binary endpoint of omission of surgery (yes vs no) with the main effect terms for Medicaid expansion status (non-expansion vs expansion), time (pre- vs post-expansion period) and the interaction term of the 2 variables. This interaction term describes the percentage point change associated with Medicaid expansion from the pre- to post-expansion period, while controlling for contemporaneous temporal change in the non-expansion states. Proportions of patients not undergoing surgery by year of diagnosis were described graphically over the entire study period. The DID parallel trends assumption was evaluated for patients diagnosed before 2014 (Supplemental Figure 1, http://links.lww.com/AOSO/A159). The analyses were repeated with adjustment for age at diagnosis, race, insurance status, comorbidity score, income, education, geographic location, facility type, and location. We also evaluated whether patient and facility factors modified the effect of Medicaid expansion, one factor at a time, by including a 3-way interaction term in the linear regression model. We then included any 3-way interaction terms with a *P* value <0.1 in a multivariate linear model. A 3-way interaction term stayed in the final multivariate model if the corresponding *P* value was <0.05. Subgroup analyses were performed to show the modified effect of Medicaid expansion by level of a patient or facility factor in the presence of a 3-way interaction. Missing data were excluded from the analyses; specifically, in the multivariate models, only complete cases who had no missing values on any of the variables (dependent variable and covariates) were included. SAS version 9.4 and S-Plus version 8.04, TIBCO Software Inc (SAS Inc, Cary, NC) are used to carry out the computations for all analyses.

## RESULTS

Of 624,237 patients diagnosed with invasive breast cancer who met the criteria for inclusion in our analytic cohort, 342,465 (54.9%) resided in non-expansion states and 281,771 (45.1%) resided in states that underwent Medicaid expansion in January 2014. Patient demographics and treatment characteristics stratified by expansion status in the pre-expansion (2010–2013) and post-expansion (2014–2017) period are summarized in Table 1. Prior to January 2014, non-expansion states had a higher proportion of patients with a median income <$30,000/y (12.5% vs 8.5%), a higher proportion of patients residing in rural communities (2.5% vs 1.2%), and fewer patients residing in metropolitan communities (80.0% vs 84.1%) as compared to expansion states (all *P* < 0.0001). Non-expansions states were overwhelmingly located in the Southern United States (79.1%) as compared to a broader geographic distribution for expansion states. Similar differences remained in the post-Medicaid expansion period (2014–2017).

**TABLE 1.** Comparison of Patient and Facility Characteristics Between Non-Expansion and Expansion States in the Pre-Medicaid Expansion (2010–2013) and Post-Medicaid Expansion (2014–2017) Period

Characteristic	Pre-Medicaid Expansion (2010–2013)					Post-Medicaid Expansion (2014–2017)				
	Non-Expansion States N = 155,839		Jan 2014 Expansion States N = 129,153		*P*	Non-Expansion States N = 186,626		Jan 2014 Expansion States N = 152,619		*P*
	N	%	N	%		N	%	N	%	
Year of diagnosis										
2010	35,438	22.7	29,075	22.5	0.0142	44,665	23.9	37,023	24.3	0.0107
2011	38,133	24.5	31,520	24.4		45,938	24.6	37,919	24.8	
2012	40,015	25.7	32,855	25.4		48,344	25.9	38,959	25.5	
2013	42,253	27.1	35,703	27.6		47,679	25.5	38,718	25.4	
Race										
Black	22,346	14.3	12,362	9.6	<0.0001	27,962	15.0	15,126	9.9	<0.0001
White	128,363	82.4	108,934	84.3		150,783	80.8	126,736	83.0	
Other	4131	2.7	6399	5.0		6361	3.4	9275	6.1	
Unknown	999	0.6	1458	1.1		1520	0.8	1482	1.0	
Charlson-Deyo Comorbidity Index										
0	126,849	81.4	106,789	82.7	<0.0001	152,775	81.9	126,305	82.8	<0.0001
1	23,335	15	17,724	13.7		25,442	13.6	19,577	12.8	
2	4356	2.8	3534	2.7		5741	3.1	4523	3.0	
3	1299	0.8	1106	0.9		2668	1.4	2214	1.5	
Median income										
<$30,000	19,520	12.5	10,997	8.5	<0.0001	21,678	11.6	12,803	8.4	<0.0001
$30,000–$34,999	28,566	18.3	15,317	11.9		30,806	16.5	17,413	11.4	
$35,000–$45,999	39,724	25.5	29,058	22.5		44,551	23.9	33,423	21.9	
$46,000+	49,316	31.6	57,363	44.4		58,406	31.3	64,357	42.2	
Unknown	18,713	12	16,418	12.7		31,185	16.7	24,623	16.1	
Education (percentage of patients with no high school degree)										
29+	26,692	17.1	12,145	9.4	<0.0001	30,180	16.2	14,405	9.4	<0.0001
20%–28.9%	33,746	21.7	22,880	17.7		37,673	20.2	25,511	16.7	
14%–19.9%	28,278	18.1	27,603	21.4		31,950	17.1	31,396	20.6	
<14%	48,394	31.1	50,080	38.8		55,623	29.8	56,661	37.1	
Unknown	18,729	12	16,445	12.7		31,200	16.7	24,646	16.1	
Insurance										
Not insured	4725	3	2003	1.6	<0.0001	4709	2.5	1317	0.9	<0.0001
Private insurance	78,812	50.6	69,230	53.6		93,923	50.3	78,434	51.4	
Medicaid	9074	5.8	8585	6.6		8613	4.6	11,861	7.8	
Medicare	60,672	38.9	48,445	37.5		76,131	40.8	59,759	39.2	
Other government	2556	1.6	890	0.7		3250	1.7	1248	0.8	
Patient’s residential location										
Metro	124,606	80	108,589	84.1	<0.0001	150,096	80.4	128,728	84.3	<0.0001
Urban	23,888	15.3	15,677	12.1		28,324	15.2	18,955	12.4	
Rural	3914	2.5	1509	1.2		4596	2.5	1816	1.2	
Unknown	3431	2.2	3378	2.6		3610	1.9	3120	2.0	
Facility type										
Academic/research program	34,208	22	48,331	37.4	<0.0001	44,963	24.1	61,592	40.4	<0.0001
Community Cancer Program	42,528	27.3	28,024	21.7		50,556	27.1	31,134	20.4	
Comprehensive Community Cancer Program	79,103	50.8	52,798	40.9		91,107	48.8	59,893	39.2	
Facility location										
Midwest	25,718	16.5	48,019	37.2	<0.0001	30,172	16.2	54,658	35.8	<0.0001
Northeast	2370	1.5	37,985	29.4		3251	1.7	46,258	30.3	
South	123,267	71.9	21,825	16.9		147,724	79.2	25,523	16.7	
West	4484	2.9	21,324	16.5		5479	2.9	26,180	17.2	
Surgery										
No	5611	3.6	4408	3.4	0.0068	8370	4.5	6339	4.2	<0.0001
Yes	150,228	96.4	124,745	96.6		178,256	95.5	146,280	95.8	

**FIGURE 2.**
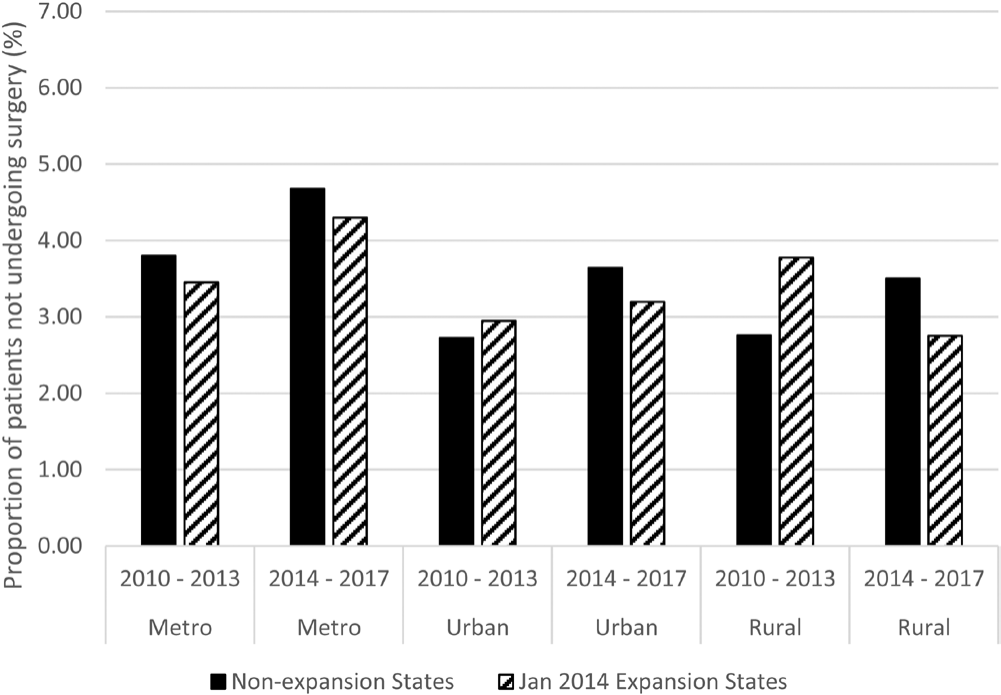
Proportions of patients not undergoing surgery over time by Medicaid expansion status and residential location.

During the overall period of study, approximately 4% of patients had omission of surgical treatment for their breast cancer. Overall, a small increase in omission of surgery was seen when comparing pre- and post-periods of study (Fig. 2) in both expansion and non-expansion states. When evaluating changes in percentages of patients who had omission of surgery by expansion status in the entire study sample, there were no significant differences between expansion states and non-expansion states in unadjusted (DID −0.14%, 95% confidence interval [CI] −0.45% to 0.05%; *P* = 0.148) or adjusted (DID −0.19%, 95% CI −0.4% to 0.03%; *P* = 0.092) DID analyses (Table 2).

**TABLE 2.** Difference-in-Differences Analysis of Breast Cancer Patients With Omission of Surgery From the Pre- to Post-Medicaid Expansion Period by Medicaid Expansion Status for the Entire Cohort and Stratified by Patient’s Residential Location

Location	Non-Expansion states			Expansion states			Difference-in-Difference Analysis			
	Pre(N = 155,839)	Post(N = 186,626)	Absolute Percent Point Change (95% CI)	Pre(N = 129,153)	Post(N = 152,619)	Absolute Percent Point Change (95% CI)	Unadjusted Percentage Point Change (95% CI)		Adjusted Percentage Point Change (95% CI)	
							% (95% CI)	*P*	% (95% CI)	*P*
Overall	5611 (3.6%)	8370 (4.48%)	0.88% (0.75%–1.02%)	4408 (3.41%)	6339 (4.15%)	0.74% (0.6%–0.88%)	−0.14% (−0.34% to 0.05%)	0.1483	−0.19% (−0.4% to 0.03%)	0.0915
Metro	4735 (3.8%)	7025 (4.7%)	0.88% (0.73%–1.03%)	3749 (3.5%)	5536 (4.3%)	0.85% (0.69%–1.01%)	−0.03% (−0.25% to 0.19%)	0.7725	−0.08% (−0.32% to 0.16%)	0.5117
Urban	651 (2.7%)	1032 (3.6%)	0.92% (0.62%–1.22%)	462 (2.9%)	606 (3.2%)	0.25% (−0.12% to 0.62%)	−0.67% (−1.15% to −0.19%)	0.0061	−0.72% (−1.25% to −0.20%)	**0.0071**
Rural	108 (2.8%)	161 (3.5%)	0.74% (0%–1.49%)	57 (3.8%)	50 (2.8%)	−1.02% (−2.22% to 0.17%)	−1.77% (−3.18% to −0.36%)	0.0141	−2.47% (−4.01% to −0.94%)	**0.0016**

The bold values indicates statistically significant differences.

However, the effect of Medicaid expansion on rates of omission of surgery varied based on a patient’s residential location (Fig. 2; Table 2). In rural areas, changes in the percentage of patients with omission of surgery were significantly different between the expansion states and the non-expansion states in unadjusted (DID −1.77%, 95% CI −3.18% to −0.36%; *P* = 0.014) and adjusted analysis (DID −2.47%, 95% CI −4.01% to −0.94%; *P* = 0.002). Specifically, the percentage of patients with omission of surgery increased from the pre-expansion to post-expansion period in the non-expansion states but decreased in the expansion states. Trends in receipt of surgery over time also differed between non-expansion and expansion states in urban areas. The percentage of patients with omission of surgery increased from the pre-expansion to post-expansion period for both expansion status groups, but the relative increase in patients with omission of surgery was less in the expansion states compared to the non-expansion states in both unadjusted (DID –0.67, 95% CI −1.15% to −0.19%; *P* = 0.006) and adjusted analyses (DID −0.72%, 95% CI −1.25% to −0.20%; *P* = 0.007). In contrast, no significant differences were seen in patients residing in metro areas (Table 2). No other patient or facility factors impacted the effect of Medicaid expansion on rates of surgery omission.

**FIGURE 3.**
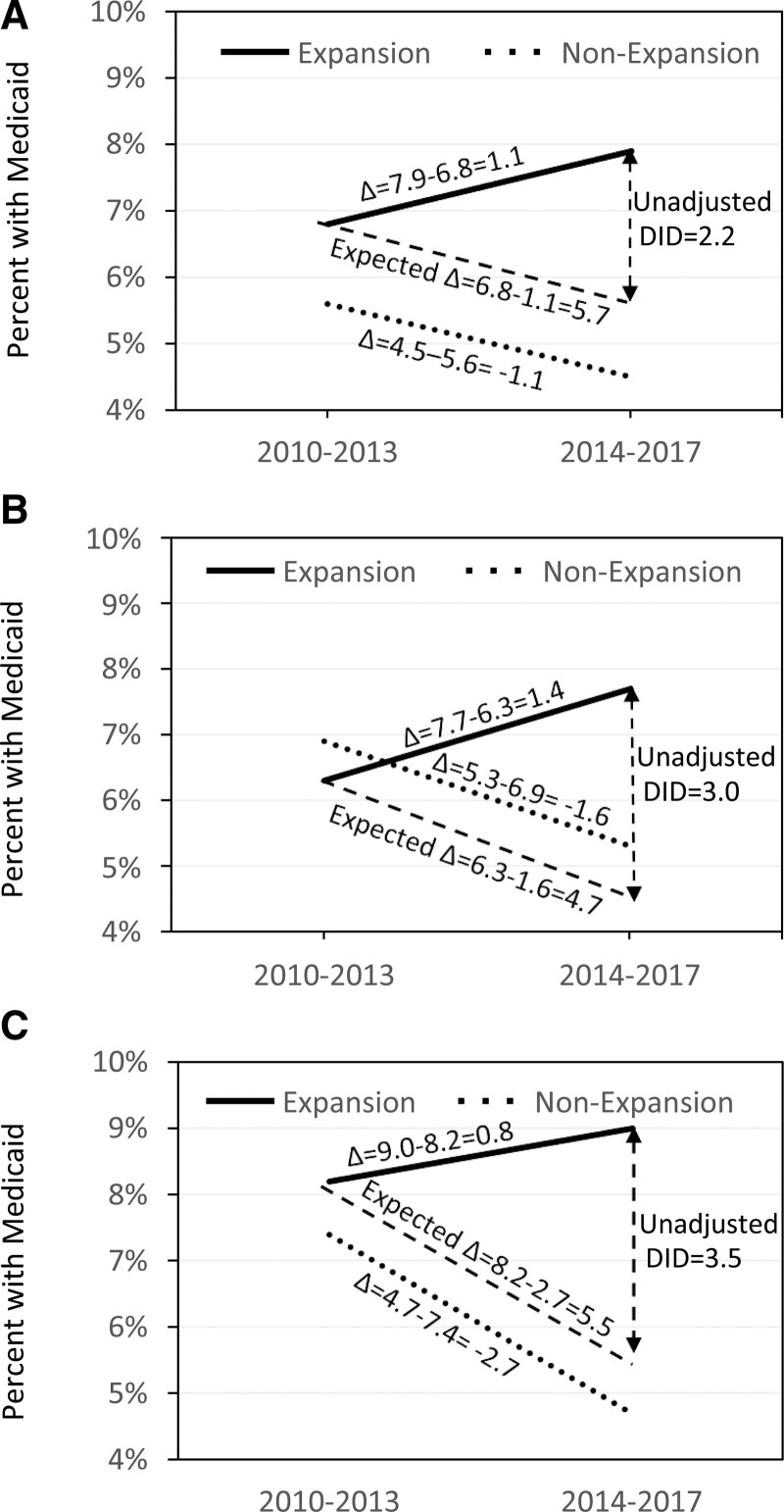
Depiction of the DID approach stratified by metro (A), urban (B), and rural (C) populations. The plot displays the change in percentage of patients with Medicaid from the pre-expansion (2010–2013) to post-expansion (2014–2017) time periods for non-expansion and expansion states.

As expected, states with Medicaid expansion had an increase in the proportion of their population covered by Medicaid, however the magnitude of increase attributable to Medicaid coverage varied by residential location (Fig. 3). Within non-expansion states, the proportion of patients covered by Medicaid declined from 5.6% to 4.5% in metro communities, 6.9% to 5.3% in urban counties, and 7.4% to 4.7% in rural communities for a net reduction of −1.1%, −1.6%, and −2.7%, respectively. Based on this, the rates of Medicaid coverage in expansion states would have been expected to decline from 6.8% to 5.7%, 6.3% to 4.7%, and 8.2% to 5.5% in metro, urban, and rural populations respectively, had Medicaid not been expanded. However, the rates of Medicaid coverage increased in all 3 areas, resulting in a 2.2% increase in Medicaid coverage attributable to Medicaid expansion in metro areas (Fig. 3A), a 3.0% increase in urban areas (Fig. 3B) and a 3.5% increase in rural areas (Fig. 3C).

## DISCUSSION

To our knowledge, our work represents the first analysis of the impact of Medicaid expansion on the surgical management of breast cancer patients. Our data demonstrate that while there was no significant difference between the rates of receipt of surgery from pre-expansion to post-expansion in expansion states and non-expansion states in the cohort of breast cancer patients as a whole, the story is more nuanced, and patients’ residential location played a significant role in the impact of Medicaid expansion under the ACA.

Our findings demonstrating no difference in overall rates of surgery in our breast cancer cohort align with a parallel hospital-based analysis in the management of colon cancer by Hoehn et al.^5^ In this study of patients with stage I to III colon cancer, Medicaid expansion did not significantly affect overall rates of operative management. The authors instead noted that Medicaid expansion impacted the characteristics of operative management, including a decline in urgent operations and a higher rate of minimally invasive surgery. In the management of breast cancer, urgent operations are rarely indicated, and thus access to surgical care becomes a more relevant surrogate to understanding effect of ACA on surgical management of breast cancer. We did note that there was an increase in omission of surgery during the period of study, though absolute differences in rates of omission were small. Despite overall out-of-pocket and premium contributions decreasing after implementation of the ACA,^15^ increases in the average annual deductibles were noted in Preferred Provider Organization health plans, which represents the most common insurance coverage for workers. Furthermore, the average deductible for covered workers has increased 153% since 2006.^16^ The increased out-of-pocket expense seen with commercial carriers may have attributed to the marginal increase rate in omission of surgery seen during the study period.

Our analysis noted a significant difference in rates of omission of surgery based on a patient’s residential location. Our findings align with previous data that indicates that a significant benefit of Medicaid expansion under the ACA was seen in rural areas.^17–20^ In our cohort of patients with invasive breast cancer residing in rural areas, Medicaid expansion was associated with fewer patients not receiving surgical management in the post-expansion period compared to an increase in omission of surgery in non-expansion states. Rural regions have a higher proportion of uninsured individuals when compared to metro locations.^21,22^ A large proportion of uninsured patients leads to poor access to care at the patient level and uncompensated care leading to financial strain, lower operating margins and risk of closure at the systems level.^23,24^

In an analysis of Medicare Hospital Cost Reports evaluating the impact of Medicaid expansion under the ACA, authors noted a decrease in uncompensated costs in states that expanded Medicaid, with the greatest reductions occurring in states with the highest levels of pre-expansion uncompensated care.^24^ It follows that a significant impact might be seen in rural hospitals, which are more heavily reliant on public funding and treat a higher proportion of uninsured patients. In fact, in a study by Lindrooth et al,^19^ authors noted fewer hospital closures in states that expanded Medicaid when compared to non-expansion states and also noted lower rates of closures seen in rural markets. Given the low population density in rural areas, maintenance of healthcare facilities is a critical issue that impacts access to and delivery of care. The enhanced impact of Medicaid expansion on rural markets, leading to greater insurance gains, a reduction in uncompensated care and fewer hospital closures provides a mechanism for our finding of a significant impact of Medicaid expansion on omission of surgery in rural areas. This is supported by the fact that our DID analytic method demonstrated the largest gain in Medicaid coverage after Medicaid expansion in rural areas. Furthermore, we noted a greater absolute reduction in the uninsured population in expansion states when compared to non-expansion states in rural areas (Supplemental Figure 2.c, http://links.lww.com/AOSO/A159).

In contrast to most of the literature on urban-rural disparities in care, the NCDB provides a 3-tier system for allocation of residence: metropolitan, urban, and rural, with urban populations including those with populations of 2500 to 20,000 individuals. Thus, the NCDB definition of urban likely includes many areas that would otherwise be designated as rural when using the Rural-Urban Commuting Area codes to dichotomize between urban and rural communities. The inclusion of rural communities within the NCDB classification of urban likely explains our finding that while the effect of Medicaid expansion in urban areas is in the same direction as in rural areas, the magnitude was not as big.

Our analysis has several limitations. As with any large, multi-institutional database, NCDB data are subject to inaccuracies in reporting. Furthermore, though the NCDB contains approximately 70% of new cancer diagnoses, this represents only 30% of hospitals in the United States that are accredited by the CoC, which could introduce an element of selection bias and limit the generalizability of our results. Given the use of hospital-level data, we also lack the rationale for omission of surgery. In patients with invasive breast cancer, surgery is offered as standard of care, and omission of surgery in the treatment algorithm is assumed to be impacted by access to and cost of care. While our inclusion criteria selected for patients with operable disease by excluding in situ and metastatic disease, our sample could have included a small proportion of patients who were not operative candidates or declined surgery for other reasons. In clinical practice, given the very small proportion of patients with non-metastatic breast cancer who are not deemed operative candidates, we do not think this significantly impacted our findings. Finally, though we used a quasi-experimental, DID approach to analysis, it is important to note that we can only identify associations between Medicaid expansion and our outcome of omission of surgery rather than making causal inferences. Furthermore, we acknowledge that there are unmeasured covariates that could systematically differ between hospitals in expansion states and non-expansion states. Despite these limitations, the study expands current knowledge by providing insight into the impact of Medicaid expansion on utilization of breast surgery for breast cancer.

In conclusion, Medicaid expansion under the ACA had a small but significant impact on rates of surgical management for breast cancer patients residing in rural and urban areas outside of large metropolitan locations, which represents a combined 15% of our study population. For the majority of the cohort, Medicaid expansion was not associated with improvement in rates of receipt of surgery. Our findings highlight that Medicaid expansion had an impact on those most disadvantaged by lack of health care access, but a minimal impact broadly with respect to overall rates of surgical management of breast cancer. While access to insurance is critically important, our findings underscore that other barriers to care will be equally important to understand in order to fully address the omission of surgery in otherwise operable and curable breast cancer.

## ACKNOWLEDGMENTS

Leisha C. Elmore, Meng Li, Yu Shen, and Isabelle Bedrosian participated in research design; Leisha C. Elmore, Meng Li, Heather Lin, Yu Shen, Simona F. Shaitelman, Gildy Babiera, Nina Tamirisa, and Isabelle Bedrosian participated in the writing of the paper; Meng Li, Heather Lin, and Yu Shen contributed new reagents or analytic tools; Leisha C. Elmore, Meng Li, Heather Lin, Yu Shen, Simona F. Shaitelman, Gildy Babiera, Nina Tamirisa, Isabelle Bedrosian participated in data analysis.

## Supplementary Material


