# Supplementary material for: Impact of Medicaid Expansion Under the Affordable Care Act on Receipt of Surgery for Breast Cancer
Source: Ann Surg Open. 2022 Aug 24;3(3):e194. doi: 10.1097/AS9.0000000000000194 (PMC9508982; doi:10.1097/AS9.0000000000000194)
Supplement: Supplementary file 1 [file as9-3-e194-s001.pdf]

SUPPLEMENTAL MATERIAL (ONLINE-ONLY FIGURES)

eFigure 1. Percentage of patients with omission of surgery for treatment of breast cancer over time for non-Medicaid expansion states and January 2014 Medicaid expansion states.

eFigure2. Distribution of patients by insurance status and residing in (A)metro, (B) urban and (C) rural areas.

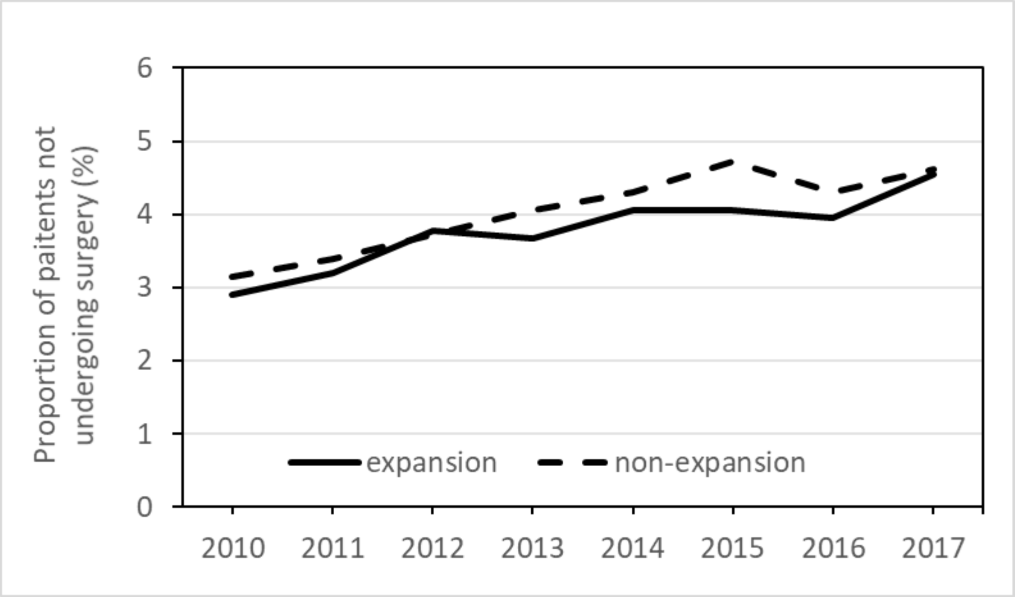

eFigure 1. Percentage of patients with omission of surgery for treatment of breast cancer over time for non-Medicaid expansion states and January 2014 Medicaid expansion states.

eFigure2. Distribution of patients by insurance status and residing in (A)metro, (B) urban and (C) rural areas.

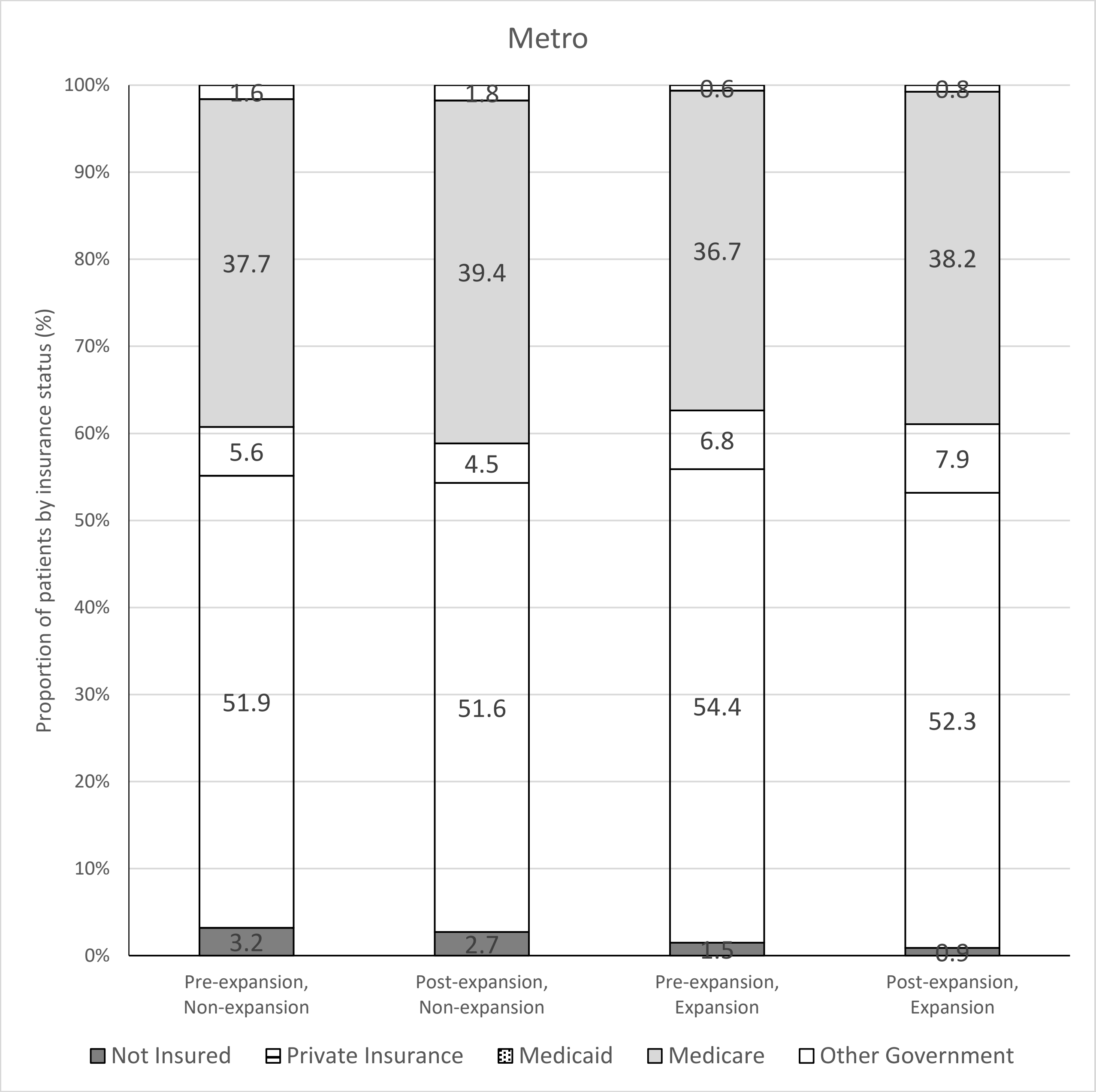

Figure 2A

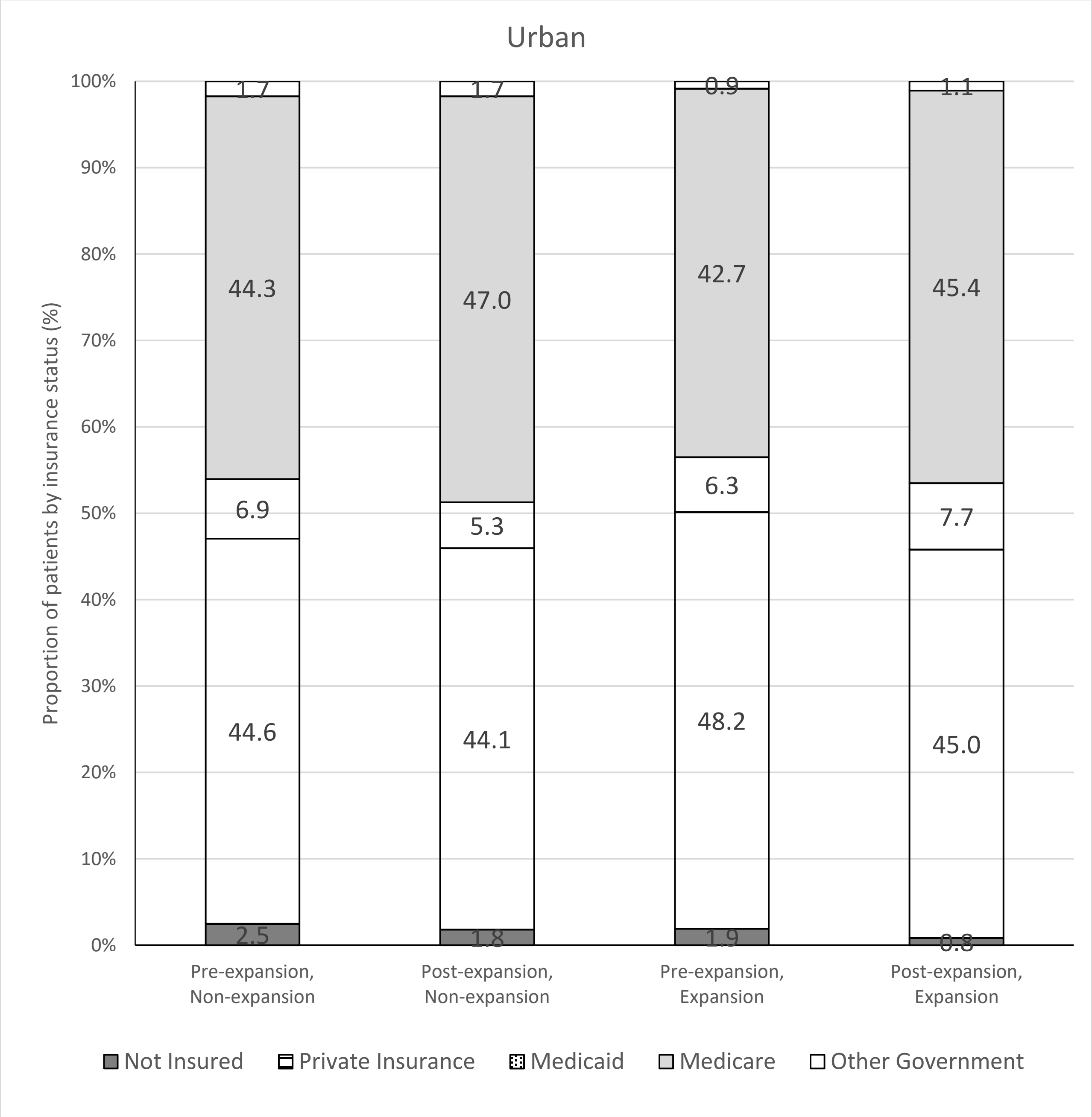
Urban
Proportion of patients by insurance status (%)
100%
90%
80%
70%
60%
50%
40%
30%
20%
10%
0%
1.7
44.3
6.9
44.6
2.5
1.7
47.0
5.3
44.1
1.8
0.9
42.7
6.3
48.2
1.9
1.1
45.4
7.7
45.0
0.8
Pre-expansion, Non-expansion
Post-expansion, Non-expansion
Pre-expansion, Expansion
Post-expansion, Expansion
Not Insured
Private Insurance
Medicaid
Medicare
Other Government

eFigure 2B

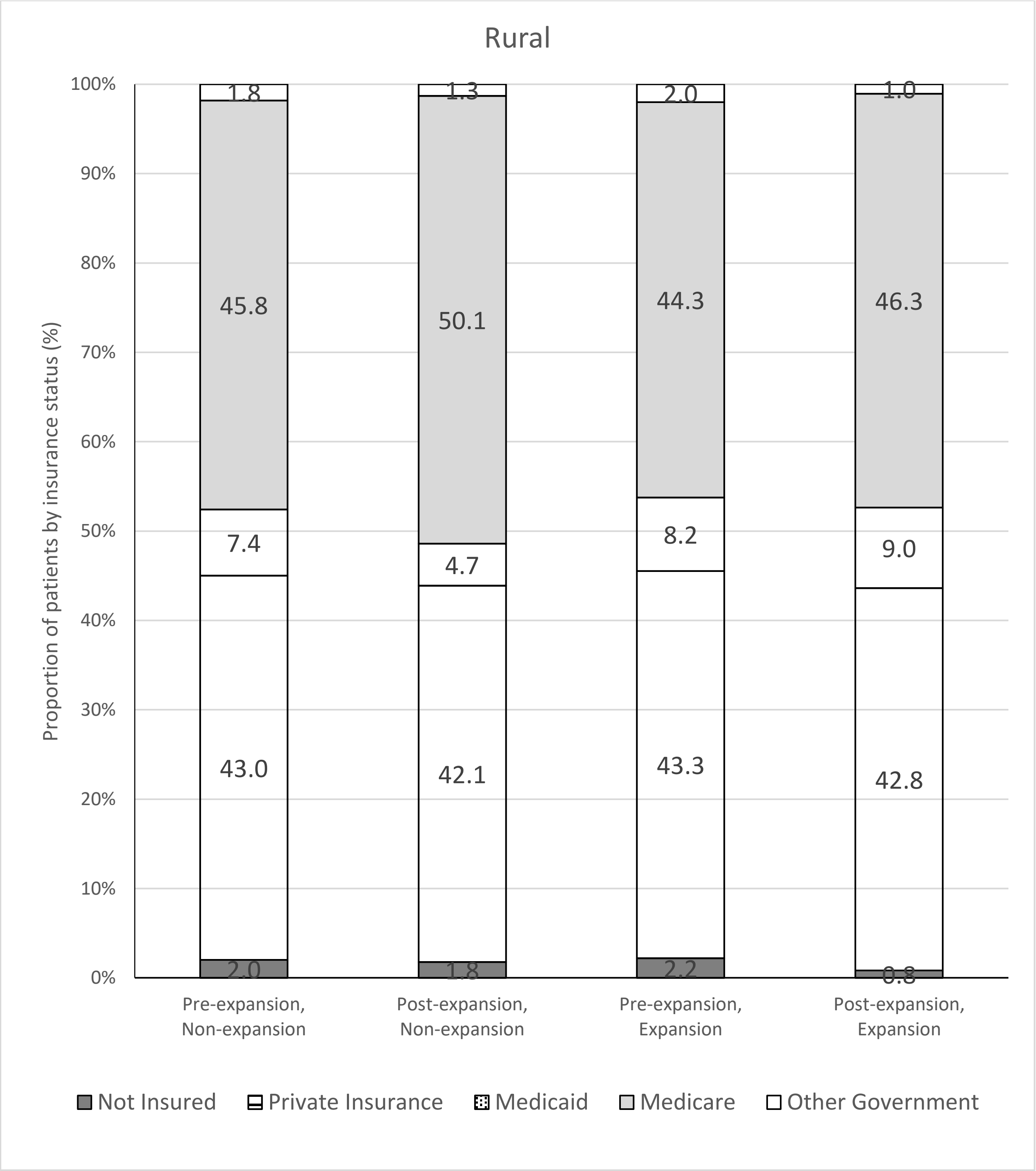

Rural
100%
90%
80%
70%
60%
50%
40%
30%
20%
10%
0%
Proportion of patients by insurance status (%)
1.8
45.8
7.4
43.0
2.0
1.3
50.1
4.7
42.1
1.8
2.0
44.3
8.2
43.3
2.2
1.0
46.3
9.0
42.8
0.8
Pre-expansion, Non-expansion
Post-expansion, Non-expansion
Pre-expansion, Expansion
Post-expansion, Expansion
Not Insured
Private Insurance
Medicaid
Medicare
Other Government

eFigure 2C
